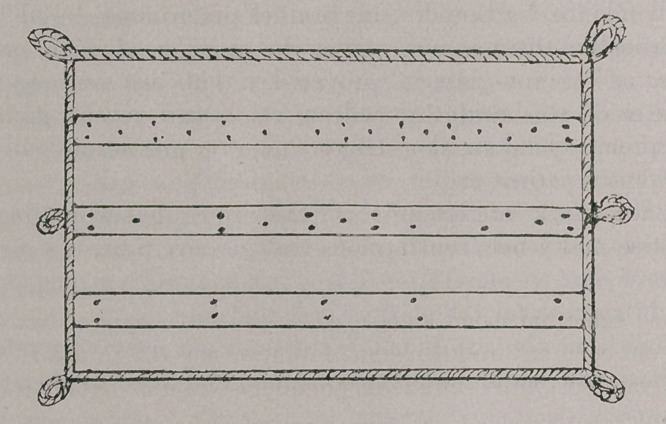# A Contrivance for the Ready Handling of Disabled Horses

**Published:** 1902-01

**Authors:** Frederick Griffith

**Affiliations:** New York City, Fellow of the New York Academy of Medicine


### A CONTRIVANCE FOR THE READY HANDLING OF DISABLED HORSES.

By Frederick Griffith,
NEW YORK CITY,
FELLOW OF THE NEW YORK ACADEMY OF MEDICINE.

Until automotive power becomes universally adopted as a means of propulsion, any appliance which will further the welfare of the horse is worthy of notice.

Having witnessed in a large city's traffic, many times, the struggles of a horse to regain his feet, sometimes totally ineffectual, save as to numberless sprains and bruises received in efforts to rise upon icy asphalt streets; also during summer's heated term, when this faithful toiler falls in the shafts from heat-stroke, the writer has to suggest a contrivance for the ready handling of the disabled animal. A strong canvas blanket is to be constructed of heavy duck cloth, cut to the size of the ordinary blanket cover; bound around with manila-rope, as is the fashion in sail making, at the four corners, and midway in the ends are to be worked eyelets or loops of rope fitted with metal thimbles for durability, for the attachment of rope, the reins, or trace chains, when in use for a disabled horse.

To strengthen the canvas where the several cloths are joined, bands six inches wide, made of trebled canvas, are to be riveted to the blanket.

So constructed the blanket, when not put to its humane use, will readily find place on a truck or wagon as a tarpaulin.

## Figures and Tables

**Figure f1:**